# Bilateral stellate ganglion block for migraine: A case report

**DOI:** 10.1097/MD.0000000000020023

**Published:** 2020-05-01

**Authors:** Suyoung Moon, Joonhee Lee, Younghoon Jeon

**Affiliations:** a Department of Anesthesiology and Pain Medicine; b Department of Anesthesiology and Pain Medicine, School of Dentistry, Kyungpook National University, Daegu, Republic of Korea.

**Keywords:** headache, migraine, pain, stellate ganglion block, sympathetic nerve

## Abstract

**Rationale:**

Migraine is a recurrent, disabling neurovascular headache disorder. The patient's quality of life can be severely impaired by migraine attacks. Stellate ganglion block (SGB) can be used to relieve pain in the head, neck, and upper extremities. In the present cases, we performed SGB in 2 patients with migraine that responds poorly to drugs.

**Patient concerns:**

Patients 1 and 2 suffered from chronic, bilateral pulsating headache, accompanied by nausea and vomiting. Patient 1 presented with headache of 8 on the visual analog scale (VAS), and 37 on the migraine disability assessment (MIDAS). Patient 2 reported headache of 7 on the VAS, and 32 on the MIDAS.

**Diagnosis:**

The patients were diagnosed with migraine without aura based on the International Classification of Headache Disorders version 3.

**Interventions:**

Patient 1 was treated with bilateral SGB every week for a month and then every month for 3 months. Patient 2 received bilateral SGB every 2 weeks for a month and then every month for 3 months.

**Outcomes:**

Four months after SGB, patient 1 reported pain intensity of 4 on VAS and 6 on MIDAS and patient 2 rated pain intensity of 3 on VAS, and 6 on MIDAS, respectively.

**Lessons:**

The SGB can be an effective option to improve headache-related disability and relieve pain intensity in the patients with refractory migraine.

## 1 Introduction

Migraine is a common, recurrent, disabling neurovascular disorder initiated by many trigger factors, such as stress, sleep deprivation, hunger, prolonged sensory stimulation, and hormonal variation.^[1]^ Until now, the exact neurovascular pathogenesis of migraine remains unclear. It was reported that migraine headache depends on an altered excitability or dysfunction of the brain state by the activation of the trigeminovascular pathway.^[2]^ Migraine is typically a pulsating and throbbing headache that can be unilateral or bilateral, accompanied by nausea, vomiting, muscle stiffness, and increased sensitivity to light, noise, smell, and touch. In addition, before a migraine attack, warning signs (premonitory symptoms) such as fatigue, mood change, food carving, and increased yawning and aura often occur; are derived from the hypothalamus, brainstem, and cortex.^[2,3]^

Patients’ quality of life can be severely impaired by migraine attacks. Migraine Disability Assessment (MIDAS) questionnaire was designed to measure headache-related disability over a 3 month period.^[4]^ The MIDAS questionnaire is a simple, self-administered questionnaire consisting of 5 questions. MIDAS has been proved to be highly reliable and correlated with clinical determination regarding the need for medical treatment.^[5]^

Stellate ganglion, a sympathetic ganglion, innervates the head, neck, and upper extremities. Stellate ganglion block (SGB) improves blood supply to the ipsilateral neck, and upper extremities, which is effective to treat vascular insufficiency.^[6,7]^ In addition, SGB is indicated for the treatment of pain disorders such as herpes zoster and complex regional pain syndrome.^[8,9]^

In the present study, we found that bilateral SGB is effective to reduce pain and improve MIDAS scores in 2 patients with migraine. Consents for the publication of this report were obtained from all patients.

## 2 Case presentation

### 2.1 Case 1

A 48-year-old female presented with a 15-year history of migraine. The headache was bilateral and pulsatile over the frontal and temporal regions, accompanied by nausea and vomiting. The frequency of pain attacks was about 3 times a month and the duration of pain was 48 hours. The pain worsened around the patient's menstrual cycles. The patient felt fatigue and dizziness 1 day before the onset of headache. The patient was diagnosed with migraine by a neurologist and was taking a daily dose of propranolol 10 mg, naproxen 500 mg, acetaminophen 1200 mg, and ergotamine 1 mg. Despite these medications, pain intensity was 8 using visual analog scale (VAS), and MIDAS score was 37 (Table 1). After informed consent was obtained, vital signs were monitored including noninvasive blood pressure, heart rate, and pulse oxygenation, and ultrasonography-guided SGB was performed by injecting 5 mL of 1.5% lidocaine at the anterior aspect of the right 6th cervical spine transverse process. Thirty minutes after the right SGB left SGB was performed with the same volume of lidocaine. No adverse effects such as hypotension, bradycardia, and dyspnea were observed. Bilateral SGB was performed once a week for a month. After the third SGB pain severity was 5 on the VAS and duration of pain was 24 hours. In addition, after the fourth SGB premonitory symptoms such as fatigue and dizziness disappeared and the pain occurred 1 day before menstruation cycle. Therefore, bilateral SGB was performed every month. Four months after bilateral SGB, MIDAS score was 6 and pain was 4 on VAS score, respectively (Table 1) and the patient was treated with oral naproxen 250 mg 2 times a day. However, migraine with pain intensity 4 on VAS continues to occur before 1 day before menstruation cycle. Therefore, patient is still treated with bilateral SGB and oral naproxen 500 mg once a month.

**Table 1:**
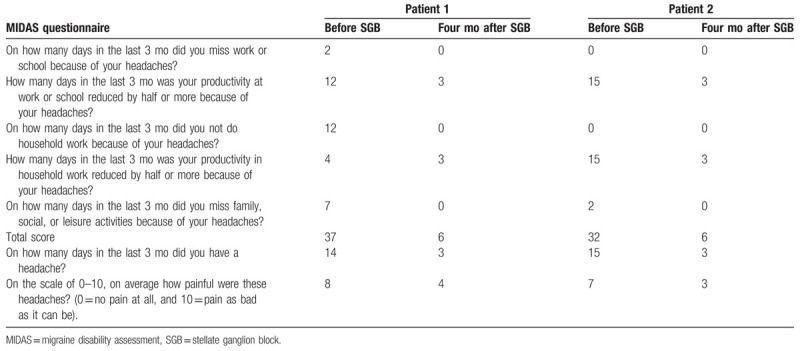
Migraine disability assessment (MIDAS) scores reported by patient 1 and 2.

### 2.2 Case 2

A 43-year-old female presented with a 10-year history of a migraine. She suffered from throbbing and pulsating headaches at bilateral temporal area, accompanied by nausea and vomiting. The headache occurred 2 to 3 times in a month and the duration of pain was 48 to 72 hours. The patient felt neck stiffness, fatigue, and depression 2 days before the onset of headache. She was managed with a combination of drugs, including acetaminophen 325 mg, dichloralphenazone 100 mg, and isometheptene 65 mg 3 times a day, as well as dexibuprofen 300 mg 3 times a day and metoclopramide 10 mg 3 times a day prescribed by a neurologist. Despite these medications, pain intensity was 7 using VAS, while the MIDAS score was 32 (Table 1). After informed consent was obtained, ultrasonography-guided SGB was performed by injecting 5 mL of 1.5% lidocaine at the anterior aspect of the right 6th cervical spine transverse process. Thirty minutes after the right SGB, the left SGB was performed. During the intervention, the patient was monitored with noninvasive blood pressure, heart rate, and pulse oxygenation, and no complications associated with SGB were observed. The patient received bilateral SGB every 2 weeks for a month. After the second bilateral SGB, the incidence and severity of migraine significantly decreased. Pain severity was 4 on the VAS score, which lasted 24 hours. In addition, premonitory symptoms such as fatigue and mood change disappeared and the pain occurred 1 day before menstruation cycle. Therefore, bilateral SGB was performed once a month. Four months after bilateral SGB, the MIDAS score was 6 and pain was 3 on VAS, respectively (Table 1). The pain was treated with oral acetaminophen 600 mg 3 times a day. However, migraine with pain intensity 3 on VAS continues to occur 1 day before menstruation cycle. Therefore, patient is still receiving bilateral SGB and oral acetaminophen 1200 mg once a month.

## 3 Discussion

Migraine is a common primary headache with a high disability and complex, hereditary neurovascular conditions arising based on functional and structural abnormality of the brain.^[1]^ Migraine predominantly affects females.^[10]^ Although the exact mechanism of migraine remains unclear, it has been suggested that abnormal activation and sensitization of trigeminovascular system play an important role in migraine pathology. Several kinds of neuropeptides, such as calcitonin gene-related peptide, nitric oxide, serotonin, and glutamate, involved in the pathogenesis of migraine.^[11–13]^

The diagnostic criteria for migraine without aura of the third edition of the international classification of headache disorders-3 includes 5 times of migraine attack with following conditions; unilateral, pulsating, moderate or severe pain, pain aggravation by or causing avoidance of routine physical activity. In addition, headache last 4 to 72 hours, companied with nausea and/or vomiting, or photophobia and phonophobia.^[14]^ The headache of patient 1 and 2 is compatible with diagnostic criteria for migraine without aura defined by the international classification of headache disorders-3. Some patients with migraine suffer from premonitory symptoms several hours to days before the onset of the headache. The premonitory symptoms are defined as the presence of nonpainful symptomatology, including fatigue, mood change, food carving, and increased yawning and neck stiffness, which may be hypothalamically modulated.^[2,3]^

Migraine can severely impact patient's quality of life. The MIDAS questionnaire assesses headache-related disability over 3 month period. This is regarded as a useful tool to improve communication between physicians and patients and to identify headache patients with significant treatment needs.^[4]^ The MIDAS score is based on 5 disability questions in 3 domains of activity (namely, office, housework, and leisure). It is considered that the patients with MIDAS score 6 or higher need medical care.^[5]^ Many drugs, including beta blockers, antidepressants, nonsteroidal anti-inflammatory drug, ergotamine, and triptan have been used for prevention or management of migraine. However, these medications cannot often alleviate migraine.^[13]^ In the present case, despite of medications, the MIDAS scores of patient 1 and 2 were 30 and 37, respectively.

The stellate ganglion, a sympathetic ganglion innervating the head, neck, and upper extremities, extensively connects with the cerebral cortex, hypothalamus, amygdala, and hippocampus.^[15]^ SGB produces several actions. First, it increases blood supply by sympatholytic effect in its sphere of innervation.^[6,7]^ Second, SGB inhibits neuronal connections between the stellate ganglion and the brain, which may produce therapeutic effects in various diseases, such as post-herpetic neuralgia, post-traumatic stress disorder, hot flashes and sleep disturbances in breast cancer survivors.^[8,16,17]^ In addition, SGB is reported to be effective for treatment of tension headache and headache following post-traumatic stress disorder.^[18,19]^ The exact mechanism of analgesic action of SGB in these disorders remains unclear. SGB decreases electroencephalogram indices of arousal in animal study and has sedative effects in healthy volunteers.^[20,21]^ This action mechanism of SGB includes restoration of the normal melatonin rhythm.^[22]^ It was reported that patient with migraine have low level of melatonin. Melatonin is effective to prevent migraine by preventing nitric oxide synthesis, inhibiting calcitonin gene-related peptide release, and antagonizing glutamate-induced excitotoxicity.^[23,24]^ In addition, stress is often cited as a trigger for migraine. In response to stress, sympathetic nerve activity increases, leading to a release of several neurotransmitters associated with pathogenesis of migraine such as dopamine, and prostaglandin. For instance, the excessive dopamine level leads to nausea, vomiting and yawing and the increase of prostaglandin augments pain sensitivity and inflammation in migraine patients.^[25,26]^ In the present cases 4 months after SGB, patient 1 reported pain intensity of 4 on VAS and 6 on MIDAS and patient 2 rated pain intensity of 3 on VAS, and 6 on MIDAS, respectively. Therefore, we presumed that SGB is effective to improve migraine by restoring normal level of melatonin and decreasing sympathetic activation with response to stress in patient with migraine.

SGB can decrease hemodynamics.^[27]^ Ultrasound-guided peripheral nerve block improves the efficacy of the intervention by direct visualization of the best site for the injection and helps avoid the damage to blood vessels and nerves.^[28,29]^ In addition, the volume of the injectate can be reduced.^[28,29]^ In the present study, the left SGB was performed 30 minutes after the right SGB with lidocaine 5 mL under ultrasound-guidance and there were no side effects such as hypotension and bradycardia.

In conclusion, SGB can be an effective option to improve MIDAS scores and pain intensity in patients with refractory migraine. Further prospective controlled studies are needed to define the therapeutic role of SGB in managing migraine patients.

## Author contributions

**Data collection:** Joonhee Lee.

**Review and editing:** Younghoon Jeon.

**Writing – original draft:** Suyoung Moon.
